# Research on the energy-saving control strategy of a belt conveyor with variable belt speed based on the material flow rate

**DOI:** 10.1371/journal.pone.0227992

**Published:** 2020-01-28

**Authors:** Jianhua Ji, Changyun Miao, Xianguo Li

**Affiliations:** 1 School of Mechanical Engineering, Tiangong University, Tianjin, China; 2 Department of Information Engineering, Tianjin University Renai College, Tianjin, China; 3 Tianjin Photoelectric Detection Technology and System Key Laboratory, Tiangong University, Tianjin, China; Tianjin University, CHINA

## Abstract

Aiming at solving the problem of high energy consumption in the rated belt speed operation of a belt conveyor system when the material flow rate is reduced, the power consumption of the frequency converter, motor, and belt conveyor is analyzed, a power consumption model of the belt conveyor system is established, the relationship between the power consumption of the belt conveyor system and belt speed is obtained, and a energy-saving control strategy of the belt conveyor with variable belt speed based on the material flow rate is put forward. The energy consumption of the belt conveyor is analyzed for a practical case. Results show that the power consumption model is accurate and the control strategy effectively reduces energy consumption. The model has high application value in coal, ports, power, mine, metallurgy, chemical, and other industries.

## 1. Introduction

The belt conveyor is used for continuous transportation in modern production. It has the advantages of large capacity, long distance, low energy consumption, low freight, high efficiency, smooth operation, and convenient loading and unloading, and is suited to bulk-material transportation. It has become one of the three main industrial transportation modes along with automobiles and trains and has been widely used in coal, ports, electricity, power, mining, metallurgy, chemical, and other industries. The operation of the belt conveyor consumes much electricity. It has been reported [1] that 41% of global electricity is provided by coal-fired power plants, and coal is one of the main sources of carbon dioxide emissions worldwide. It is therefore imperative to reduce the energy consumption of the belt conveyor system.

There are two ways to reduce the energy use of the belt conveyor: one being to improve the performance of the equipment and the other being to optimize operation parameters (e.g., the belt speed). Daijie He *et al*. [2] studied the transient operation dynamics of a conveyor belt while adjusting the speed of the belt conveyor and solved the time optimization problem of the speed adjustment. Other studies [3–5] focused on the belt conveyor and proposed a power-saving model for control of the speed of the belt conveyor. Through analysis of the energy model of the belt conveyor and ring hammer crusher, Shirong Zhang *et al*. [6] proposed a power-saving model. The problem with the above models is that they do not take the belt conveyor system as the research object but only consider the energy consumption of the belt conveyor itself.

Aiming to solve the problem of high energy consumption in the rated belt speed operation of the belt conveyor when the material flow rate is reduced, the present paper analyzes the power consumption of the frequency converter, motor, and belt conveyor, establishes a power consumption model of the belt conveyor system, and proposes a energy-saving control strategy for the belt conveyor with a variable belt speed based on the material flow rate. The energy consumption of the belt conveyor system is analyzed for a practical case. Results show that the power consumption model of the system is accurate and the energy-saving control strategy achieves the expected energy savings.

## 2. Power consumption model of the belt conveyor system

**Fig 1:**
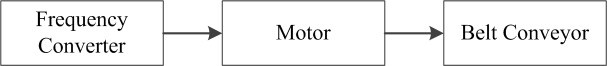
The system block diagram of the belt conveyor.

The belt conveyor system studied in this paper comprises a frequency converter, motor, and belt conveyor (Fig 1).

### 2.1 Power consumption of the belt conveyor

According to ISO 5048, when the length of the belt conveyor exceeds 80 meters or when a single conveyor has only one loading point, the resistance *F*_*U*_ of the belt conveyor is

$$\begin{array}{l} F_{U}=CF_{H}+F_{S1}+F_{S2}+F_{St}=CfLg\left[q_{RO}+q_{RU}+\left(2q_{B}+q_{G}\right)\cos\delta \right]\\ \;+\left[C_{\varepsilon }\mu_{0}L_{\varepsilon }g\left(q_{B}+q_{G}\right)\cos\delta \sin\varepsilon +\frac{1000\mu_{2}I_{V}^{2}\rho gl}{v^{2}b_{1}^{2}}\right]+\left(\sum Ap\mu_{3}+Bk_{p}\right)+Hgq_{G} \end{array},$$ (1)

where *C* is the additional-resistance coefficient, *F*_*H*_ is the main resistance, *F*_*S*1_ is the main special resistance, *F*_*S*2_ is the additional special resistance, and *F*_*St*_ is the lifting resistance.

If the belt speed of the belt conveyor is denoted *v* (unit: m/s) and the material flow rate is denoted *M* (unit: t/h), then

$$M=3.6q_{G}\cdot v,$$ (2)

where *q*_*G*_ is the linear density of the material (kg/m).

Considering Eq (2), the power consumption of the belt conveyor during stable operation is

$$\begin{array}{l} p_{BC}=\left[CfLg\left(q_{RO}+q_{RU}+2q_{B}\cos\delta \right)+C_{\varepsilon }\mu_{0}L_{\varepsilon }gq_{B}\cos\delta \sin\varepsilon +\sum Ap\mu_{3}+Bk_{p}\right]\cdot v\\ \;+\left[\frac{1000\mu_{2}I_{V}^{2}\rho gl}{b_{1}^{2}}\right]\cdot \frac{1}{v}+\left[\frac{CfLg\cos\delta }{3.6}+\frac{C_{\varepsilon }\mu_{0}L_{\varepsilon }g\cos\delta \sin\varepsilon }{3.6}+\frac{Hg}{3.6}\right]\cdot M \end{array}.$$ (3)

This equation can be decomposed to

$$\begin{array}{l} p_{BC}(v)=\left[CfLg\left(q_{RO}+q_{RU}+2q_{B}\cos\delta \right)+C_{\varepsilon }\mu_{0}L_{\varepsilon }gq_{B}\cos\delta \sin\varepsilon +\sum Ap\mu_{3}+Bk_{p}\right]\cdot v\\ \;+\left[\frac{1000\mu_{2}I_{V}^{2}\rho gl}{b_{1}^{2}}\right]\cdot \frac{1}{v} \end{array},$$ (4)

$$p_{BC}(M)=\left[\frac{CfLg\cos\delta }{3.6}+\frac{C_{\varepsilon }\mu_{0}L_{\varepsilon }g\cos\delta \sin\varepsilon }{3.6}+\frac{Hg}{3.6}\right]\cdot M.$$ (5)

Obviously, *p*_*BC*_ (*v*) only relates to the belt speed while *p*_*BC*_ (*M*) only relates to the material flow rate.

### 2.2 Power consumption of the motor

The temperature change of the motor has little effect on the resistance and inductance parameters of the stator and rotor, and the effect of the temperature change of the motor is thus not considered in the analysis of the motor loss.

**Fig 2:**
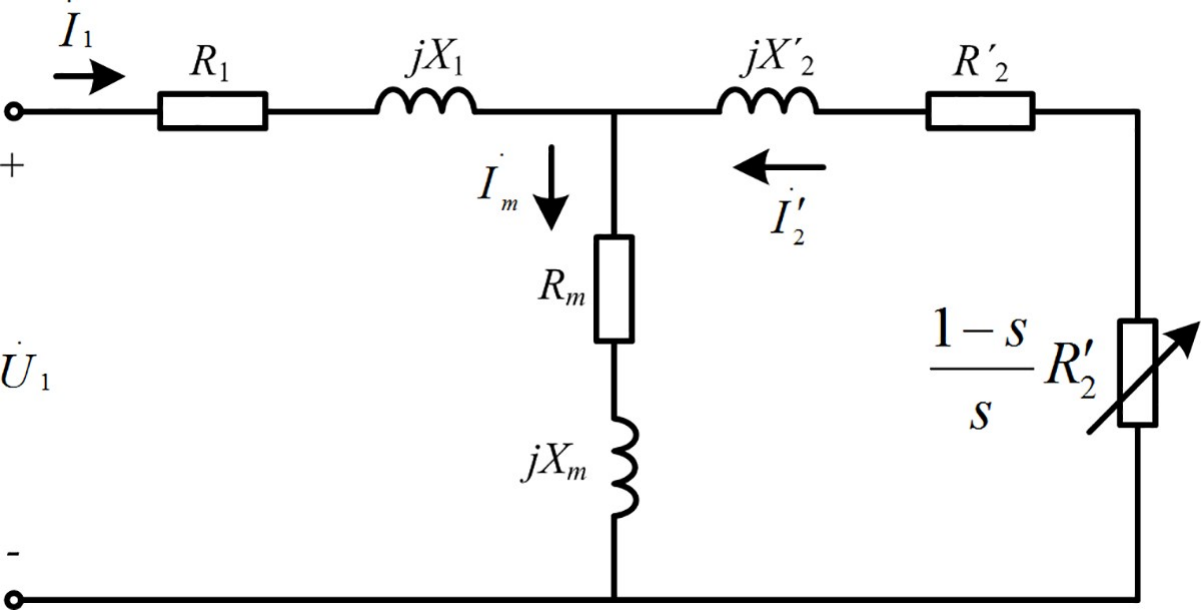
The equivalent circuit diagram of three phase asynchronous motor in stable operation.

Fig 2 is the equivalent circuit diagram of a three-phase asynchronous motor running stably at speed *n* [7]. In Fig 2, ${\dot{U}}_{1}$ is the phase voltage of the power supply, ${\dot{I}}_{1}$ is the input current of the stator, ${\dot{I}}_{m}$ is the excitation current, ${\dot{I}}_{2}^{\prime }$ is the converted rotor circuit current, *R*_1_ is the stator winding resistance, *X*_1_ is the stator leakage reactance, *R*_*m*_ is the excitation resistance, *X*_*m*_ is the excitation reactance, *R*'_2_ is the converted rotor winding resistance, *X*'_2_ is the converted rotor leakage reactance, and $\frac{1-s}{s}R_{2}^{\prime }$ is the equivalent load resistance after conversion.

The current of the equivalent circuit is calculated as

$${\dot{I}}_{1}=\frac{{\dot{U}}_{1}(z_{m}+z_{2}^{\prime })}{z_{1}z_{m}+z_{m}z_{2}^{\prime }+z_{1}z_{2}^{\prime }},$$ (6)

$${\dot{I}}_{2}^{\prime }=-\frac{{\dot{U}}_{1}z_{m}}{z_{1}z_{m}+z_{m}z_{2}^{\prime }+z_{1}z_{2}^{\prime }},$$ (7)

$${\dot{I}}_{m}=\frac{{\dot{U}}_{1}z_{2}^{\prime }}{z_{1}z_{m}+z_{m}z_{2}^{\prime }+z_{1}z_{2}^{\prime }},$$ (8)

where *z*_1_=*R*_1_+*jX*_1_, *z*_*m*_ = *R*_*m*_+*jX*_*m*_, and $z_{2}^{\prime }=R_{2}^{\prime }+\frac{1-s}{s}R_{2}^{\prime }+jX_{2}^{\prime }=\frac{1}{s}R_{2}^{\prime }+jX_{2}^{\prime }$.

The stator copper loss, iron loss, and rotor copper loss of the motor are respectively

$$p_{Cu1}=3I_{1}^{2}R_{1},$$ (9)

$$p_{Fe}=3I_{m}^{2}R_{m},$$ (10)

$$p_{Cu2}=3I_{2}^{\prime 2}R_{2}^{\prime }.$$ (11)

According to [8], the stray loss and mechanical loss account for about 20% of the total loss. The total loss of the three-phase asynchronous motor is therefore

$$p_{motor}=1.25\times \left(p_{Cu1}+p_{Fe}+p_{Cu2}\right).$$ (12)

The motor is regulated by frequency conversion through the frequency converter, and the reactance in the equivalent circuit thus changes with the frequency of the input power supply. Hence,

$$\begin{array}{l} X_{1}=2\pi f_{1}L_{1}\\ X_{m}=2\pi f_{1}L_{m}\\ X_{2}^{\prime }=2\pi f_{1}L_{2} \end{array}\},$$ (13)

where *f*_1_ is the input frequency of the motor and *L*_*x*_ is the inductance.

As a coefficient related to the motor flux, α is defined as

$$\alpha =\frac{U_{1}}{f_{1}}=4.44N_{1}k_{N1}\Phi .$$ (14)

Where *N*_1_ is the number of stator turns, *k*_*N*1_ is the winding factor and Φ is the main flux.

Under the condition of constant torque load, *N*_1_, *k*_*N*1_ and Φ are constants, so *α* is a constant. Therefore, for the same belt conveyor with constant torque load, α is a constant.

The stable operation of the belt conveyor relates to a constant torque load; i.e., *α* is a fixed value. The input voltage of the motor is therefore proportional to the frequency of the power supply:

$$U_{1}=\alpha f_{1}.$$ (15)

The relationship between the stator speed and power frequency is

$$n_{1}=\frac{60f_{1}}{p},$$ (16)

where *p* is the number of pole pairs of the motor.

The slip ratio is calculated as

$$s=\frac{n_{1}-n}{n_{1}},$$ (17)

where *n*_1_ is the synchronous speed (unit: r/min) and n is the rotor speed (unit: r/min).

Owing to the small change in the slip ratio, to simplify the calculation, it is assumed that the slip ratio is fixed and

$$f_{1}=\frac{np}{60(1-s)}.$$ (18)

It follows from the relationship between the linear speed and rotational speed that

$$n=\frac{60v}{2\pi R},$$ (19)

where *v* is the running speed of the belt conveyor and *R* is the radius of the driving drum of the belt conveyor.

The relationship between the belt speed and the frequency of the output power supply of the frequency converter is therefore

$$f_{1}=\frac{vp}{2\pi R(1-s)}.$$ (20)

The relationship between the supply phase voltage and belt speed is

$$U_{1}=\frac{\alpha vp}{2\pi R(1-s)}.$$ (21)

The relationship between the impedance and belt speed is

$$\begin{array}{l} z_{1}=R_{1}+j\frac{vpL_{1}}{R(1-s)}\\ z_{m}=R_{m}+j\frac{vpL_{m}}{R(1-s)}\\ z_{2}^{\prime }=\frac{R_{2}^{\prime }}{s}+j\frac{vpL_{2}}{R(1-s)} \end{array}\}.$$ (22)

On the basis of Eqs (6)–(11) and Eq (21), the stator copper loss, iron loss, and rotor copper loss are respectively

$$p_{Cu1}=3R_{1}{\left|\frac{\alpha vp}{2\pi R(1-s)\left(z_{1}+\frac{z_{m}z_{2}^{\prime }}{z_{m}+z_{2}^{\prime }}\right)}\right|}^{2},$$ (23)

$$p_{Fe}=3R_{m}{\left|\frac{\alpha vp-2\pi R(1-s){\dot{I}}_{1}z_{1}}{2\pi R(1-s)z_{m}}\right|}^{2},$$ (24)

$$p_{Cu2}=3R_{2}^{\prime }{\left|\frac{2\pi R(1-s){\dot{I}}_{1}z_{1}-\alpha vp}{2\pi R(1-s)z_{2}^{\prime }}\right|}^{2}.$$ (25)

The power consumption of the motor is

$$p_{motor}(v)=\frac{15\alpha^{2}p^{2}R_{1}v^{2}}{16\pi^{2}R^{2}{\left(1-s\right)}^{2}{\left|z_{1}+\frac{z_{m}z_{2}^{\prime }}{z_{m}+z_{2}^{\prime }}\right|}^{2}}+\frac{15R_{m}}{4}{\left|\frac{\alpha vp-2\pi R(1-s){\dot{I}}_{1}z_{1}}{2\pi R(1-s)z_{m}}\right|}^{2}+\frac{15R_{2}^{\prime }}{4}{\left|\frac{2\pi R(1-s){\dot{I}}_{1}z_{1}-\alpha vp}{2\pi R(1-s)z_{2}^{\prime }}\right|}^{2}.$$ (26)

Therefore, the power consumption of the motor is a function of the belt speed of the belt conveyor.

### 2.3 Power consumption of the frequency converter

The power loss of the frequency converter mainly comprises losses of the insulated-gate bipolar transistor (IGBT) and fast-recovery diode [9]. The fast-recovery diode has power loss at the moments of switching on and off but its leakage current is low when it is off, and the turn-on loss is thus negligible compared with the turn-off loss [10].

**Fig 3:**
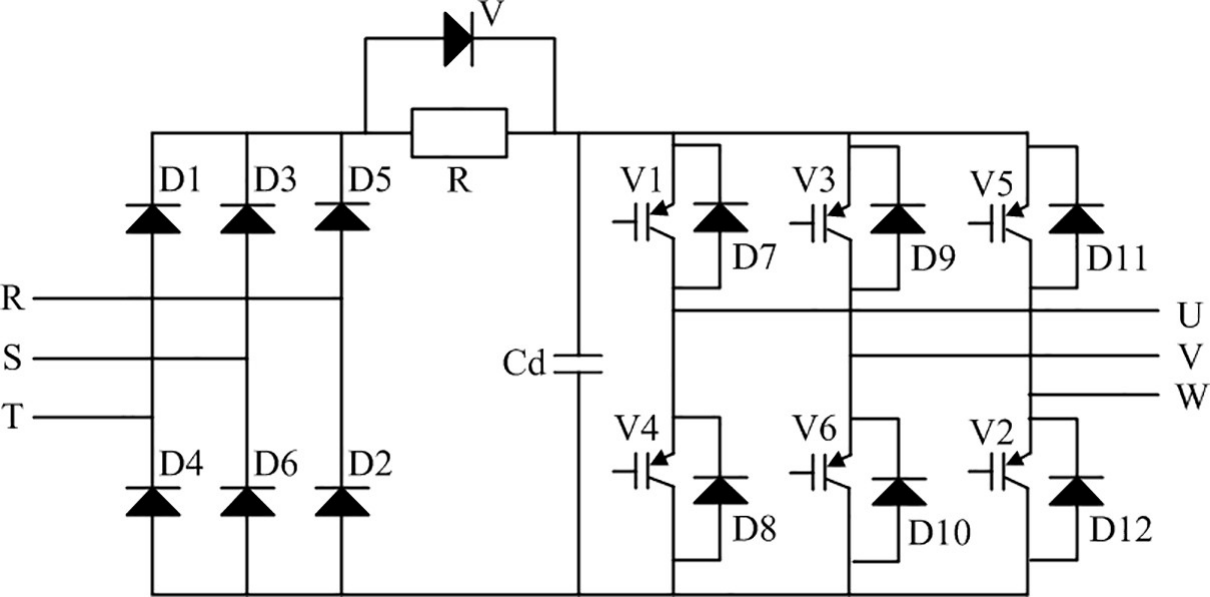
The main circuit of the frequency converter.

Fig 3 shows the main circuit of the frequency converter, including the rectifier, inverter, and intermediate circuit. The turn-on and turn-off of the inverter are controlled by the SVPWM pulse.

The on-state loss of the IGBT is [11, 12]

$$p_{fw/V}=\frac{\alpha^{2}p^{2}r_{CE}\left(\frac{3}{4}+\frac{2m\cos\phi }{\pi }\right)}{2\pi^{2}R^{2}{\left(1-s\right)}^{2}{\left|z_{1}+\frac{z_{m}z_{2}^{\prime }}{z_{m}+z_{2}^{\prime }}\right|}^{2}}v^{2}+\frac{\sqrt{2}\alpha pV_{CEO}\left(\frac{3}{\pi }+\frac{3m\cos\phi }{4}\right)}{2\pi R(1-s)\left|z_{1}+\frac{z_{m}z_{2}^{\prime }}{z_{m}+z_{2}^{\prime }}\right|}v,$$ (27)

where *m* is the modulation ratio, cos*φ* is the power factor, *r*_CE_ is the on-state resistance of the IGBT, *V*_CEO_ is the actual on-state voltage drop of the IGBT, and *I*_CM_ is the peak of effective current.

The on-state loss of the fast recovery diode is

$$p_{fw/D}=\frac{\alpha^{2}p^{2}r_{F}\left(\frac{3}{4}-\frac{2m\cos\phi }{\pi }\right)}{2\pi^{2}R^{2}{\left(1-s\right)}^{2}{\left|z_{1}+\frac{z_{m}z_{2}^{\prime }}{z_{m}+z_{2}^{\prime }}\right|}^{2}}v^{2}+\frac{\sqrt{2}\alpha pV_{FO}\left(\frac{3}{\pi }-\frac{3m\cos\phi }{4}\right)}{2\pi R(1-s)\left|z_{1}+\frac{z_{m}z_{2}^{\prime }}{z_{m}+z_{2}^{\prime }}\right|}v,$$ (28)

where *r*_F_ is the on-state resistance of the fast-recovery diode while *V*_FO_ is the actual on-state voltage drop of the diode.

The switching loss of the IGBT is

$$p_{SW}=\frac{6\sqrt{2}\alpha pf_{SW}V_{dc}\left(E_{SW(on)}+E_{SW(off)}\right)}{2\pi^{2}I_{CN}V_{CEN}R(1-s)\left|z_{1}+\frac{z_{m}z_{2}^{\prime }}{z_{m}+z_{2}^{\prime }}\right|}v,$$ (29)

where *f*_*SW*_ is the switching frequency, *V*_*dc*_ is the DC bus voltage, *V*_*CEN*_ is the rated voltage, *I*_*CN*_ is the rated current, *E*_*sw*(*on*)_ is the instantaneous energy loss of the IGBT each time that it shifts from the turned-off state to the turned-on state under the rated current *I*_*CN*_ and rated voltage *V*_*CEN*_, and *E*_*sw*(*off*)_ is the instantaneous energy loss of the IGBT each time that it shifts from the turned-on state to the turned-off state under the rated current *I*_*CN*_ and rated voltage *V*_*CEN*_.

The power consumption of the frequency converter is obtained from Eqs (27)–(29) as

$$p_{FC}(v)=p_{fw/V}+p_{fw/D}+p_{SW}=\frac{k_{FC1}}{{\left|z_{1}+\frac{z_{m}z_{2}^{\prime }}{z_{m}+z_{2}^{\prime }}\right|}^{2}}v^{2}+\frac{k_{FC2}}{\left|z_{1}+\frac{z_{m}z_{2}^{\prime }}{z_{m}+z_{2}^{\prime }}\right|}v,$$ (30)

where $k_{FC1}=\frac{\alpha^{2}p^{2}\left[\frac{3}{4}\left(r_{CE}+r_{F}\right)+\frac{2m\cos}{\pi }\left(r_{CE}-r_{F}\right)\right]}{2\pi^{2}R^{2}{\left(1-s\right)}^{2}}$ and

$$k_{FC2}=\frac{\sqrt{2}\alpha p\left[\frac{3}{\pi }\left(V_{CEO}+V_{FO}\right)-\frac{3m\cos}{4}\left(V_{CEO}-V_{FO}\right)+\frac{6f_{SW}V_{dc}\left(E_{SW(on)}+E_{SW(off)}\right)}{\pi I_{CN}V_{CEN}}\right]}{2\pi R(1-s)}.$$

It can be seen that the power consumption of the frequency converter is also a function of the belt speed of the belt conveyor.

### 2.4 Power consumption of the belt conveyor system

The total power consumption of the belt conveyor system is obtained from Eqs (3), (26) and (30) as

$$p(v,M)=p_{BC}(v,M)+p_{motor}(v)+p_{FC}(v)=p_{BC}(M)+p_{BC}(v)+p_{motor}(v)+p_{FC}(v).$$ (31)

Here, the power consumption relating to the belt speed of the belt conveyor is

$$p(v)=p_{BC}(v)+p_{motor}(v)+p_{FC}(v).$$ (32)

The power consumption of the belt conveyor system is therefore related to the belt speed and material flow rate of the belt conveyor.

## 3.Variable-belt-speed energy-saving control strategy

### 3.1 Belt speed having the greatest energy savings

Reducing the material flow rate can reduce *p*_BC_(*M*) while adjusting the belt speed can reduce *p*(*v*). Considering that reducing the material flow rate will reduce the transportation efficiency, the power consumption can be reduced by changing the belt speed.

The power saved by the belt conveyor system comes from *p*(*v*) and is independent of the material flow rate. The power savings are therefore fixed values as long as the belt speed is determined.

**Fig 4:**
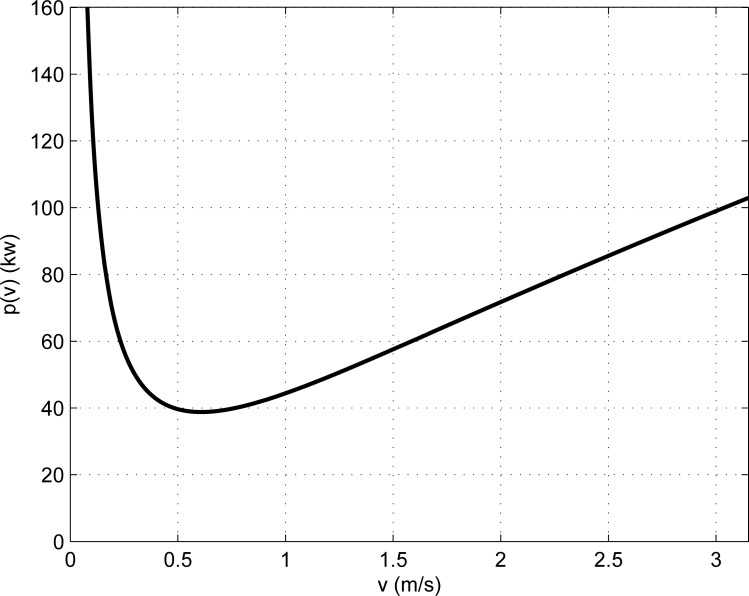
The effect of the belt speed variation on *p*(*v*).

On the basis of Eqs (4), (26), (30) and (32), *p*(*v*) can be divided into two parts, one is approximately proportional to the belt speed, and the other is approximately inversely proportional to the belt speed. Therefore, when the belt speed is relatively low, the part which is approximately inversely proportional to the belt speed plays a leading role, and at this time *p*(*v*) is approximately inversely proportional to the belt speed; when the belt speed is relatively high, the part which is approximately proportional to the belt speed plays a leading role, and at this time *p*(*v*) is approximately proportional to the belt speed. The effect of the belt speed of the belt conveyor system on *p*(*v*) is presented in Fig 4.

Fig 4 shows that when *v* > *v*_*pmin*_, *p* (*v*) is positively correlated with the belt speed of the belt conveyor. The energy-saving effect of the system is therefore more obvious when the belt speed is lower.

The belt speed of the belt conveyor system with minimum power consumption can be obtained by solving the differential equation

$$\frac{dp(v)}{dv}=0.$$ (33)

### 3.2 Avoiding material stockpiling

The belt conveyor has the best energy-saving effect when it runs at the belt speed *v*_*pmin*_, but material may accumulate at such a low speed. In ensuring the safe operation of the belt conveyor system, it is necessary to reduce the energy consumption without stockpiling.

The linear density of rated material is defined as the maximum mass of material per meter allowed by the conveyor belt and is denoted *q*_*Gm*_ (kg/m). *q*_*Gm*_ is a fixed value for the determined conveyor belt.

To ensure that the belt conveyor does not stockpile material, the belt speed of the belt conveyor must satisfy

$$v\geq \frac{M}{3.6q_{Gm}}.$$ (34)

Fig 4 shows that the belt conveyor has the best energy-saving effect without stockpiling when the equality in Eq (34) holds. In this case, the belt speed of the belt conveyor is proportional to the material flow rate. The essence of reducing the belt speed of the belt conveyor is to ensure full-load operation of the conveyor.

When the belt conveyor runs at full load, the rated belt speed of the belt conveyor is

$$v_{e}=\frac{M_{e}}{3.6q_{Gm}},$$ (35)

where *M*_*e*_ is the rated material flow rate of the belt conveyor.

### 3.3 Energy-saving control strategy

Considering the safety and energy savings of the belt conveyor comprehensively, to achieve the best energy-saving effect, the energy-saving control strategy of the belt conveyor system with variable belt speed is

$$v=\left\{ \begin{array}{l} \frac{M}{3.6q_{Gm}},3.6q_{Gm}v_{p\min}\leq M\leq M_{e}\\ v_{p\min},\;0<M<3.6q_{Gm}v_{p\min} \end{array}\right..$$ (36)

## 4. Analysis of a practical case

### 4.1 System parameters of a belt conveyor

This paper takes a belt conveyor considered in the literature [4] as the research carrier. Table 1 gives the main design parameters of the belt conveyor while Table 2 gives the main parameters of the motor and Table 3 gives the main parameters of the IGBT module.

**Table 1:** The parameters of the belt conveyor.

Parameter description	Symbol	Value	Unit
Transfer rate	*Qm*	2000	*t/h*
Surcharge angle	*θ*	30	°
Friction factor	*f*	0.024	*—*
Troughing angle	*λ*	35	°
Maximum sectional area	*A*	0.253	*m*^2^
Belt speed	*V*_*e*_	3.15	*m/s*
Unit mass of the belt	*q*_*B*_	18.73	*kg*/*m*
Inclination angle	*δ*	1.825	°
Width of the belt	*B*	1400	*mm*
Safety factor of belt	*S*_*A*_	8	*—*
Density of the material	*ρ*	900	*kg*/*m*^3^
Interval of the skirt boards	*b*_*1*_	0.85	*m*
Inclination coefficient	*k*	1.0	*—*
The net change in elevation	*H*	9.98	*m*
Main resistance factor	*C*	1.31	*—*
Friction factor between driving drum and belt	*μ*	0.3	*—*
Friction factor between belt and idlers	*μ*_0_	0.3	*—*
Fiction factor between material and belt	*μ*_1_	0.6	*—*
Fiction factor between material and skirt board	*μ*_2_	0.6	*—*
Fiction factor between belt and its cleaners	*μ*_3_	0.6	*—*
Coefficient of the scraping board	*k*_*p*_	1500	*N*/*m*
Pressure exerted on belt by belt cleaner	*p*	1×10^5^	*N*/*m*^2^
Coefficient of the troughing shape	*Cε*	0.45	*—*
Surrounding angle of conveyor belt in driving drum	*φ*	3.14	*rad*
Unit mass of rotating parts of carrying idlers	*q*_*RO*_	15.75	*kg*/*m*
Unit mass of rotating parts of return idlers	*q*_*RU*_	7.76	*kg*/*m*
Centre-to-centre distance of the belt	*L*	313.25	*m*
Length of skirt boards outside feeder station	*l*	4.5	*m*
Forwards tiling angle of idlers	*ε*	2	°

**Table 2:** The parameters of the motor.

Parameter description	Symbol	Value	Unit	Parameter description	Symbol	Value	Unit
Rated voltage	*U*	380	*V*	number of pole pairs	*p*	2	*—*
Rated current	*I*	495	*A*	Power frequency	*f*	50	*Hz*
Rated power	*P*	280	*kW*	Power factor	cos*φ*	0.9	*—*
Stator speed	*n*_*1*_	1500	*r/min*	Efficiency	*η*	95.1%	*—*
Rotor speed	*n*	1425	*r/min*				

**Table 3:** The parameters of the IGBT module.

Parameter description	Symbol	Value	Unit	Parameter description	Symbol	Value	Unit
IGBT on-state resistance	*r*_*CE*_	0.00254	*Ω*	Rated voltage	*V*_*CEN*_	1700	*V*
Diode on-state resistance	*r*_F_	0.00179	*Ω*	Rated current	*I*_*CN*_	600	*A*
IGBT on-state voltage drop	*V*_CEO_	0.75	*V*	Switching frequency	*f*_*SW*_	10	*kHz*
Diode on-state voltage drop	*V*_FO_	0.6	*V*	Power factor	cos*φ*	0.85	*—*
IGBT Single Turn-on Loss	*Esw*(*on*)	0.21	*J*	Modulation ratio	*m*	0.9	*—*
IGBT Single Turn-off Loss	*Esw*(*off*)	0.15	*J*				

### 4.2 Analysis of the energy-saving effect

**Fig 5:**
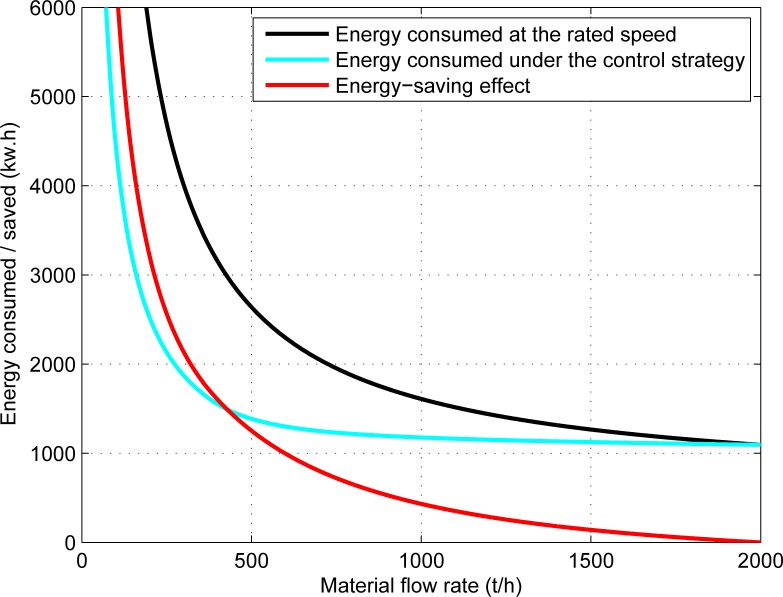
The relationship between the energy consumption (energy saving) of the system and the material flow rate.

**Fig 6:**
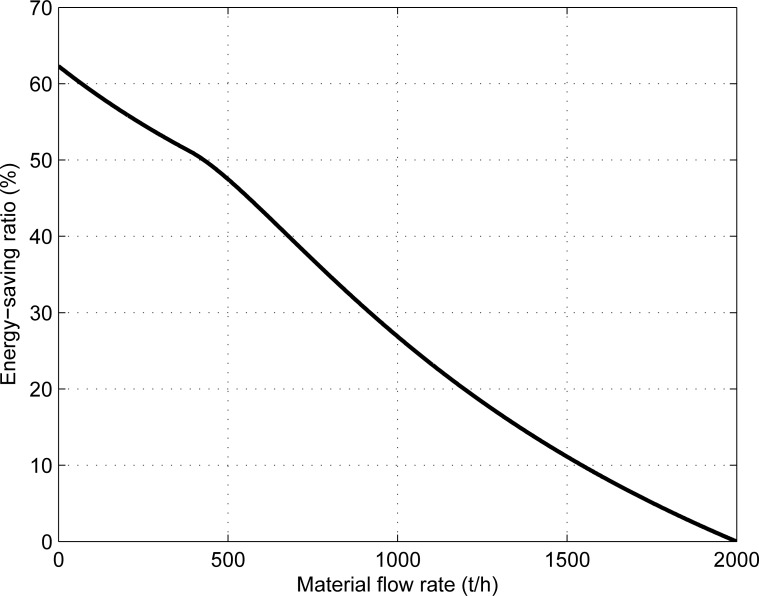
The relationship between the energy-saving ratio of the system and the material flow rate.

The energy-saving analysis is based on the condition of transporting 10000 tons of materials. The energy consumption and energy-saving effect of the belt conveyor system for different material flow rates are presented in Fig 5, and the relationship between the energy-saving ratio of the system and the material flow rate is shown in Fig 6.

Fig 5 shows that, the system does not save energy for rated material flow rate, but the energy-saving effect is more and more obvious with the decrease of material flow rate.

Fig 6 shows that the energy-saving ratio of the system increases gradually with the decrease of material flow rate and the energy saving rate can reach 62.304% at no-load, which is consistent with the theoretical analysis.

**Fig 7:**
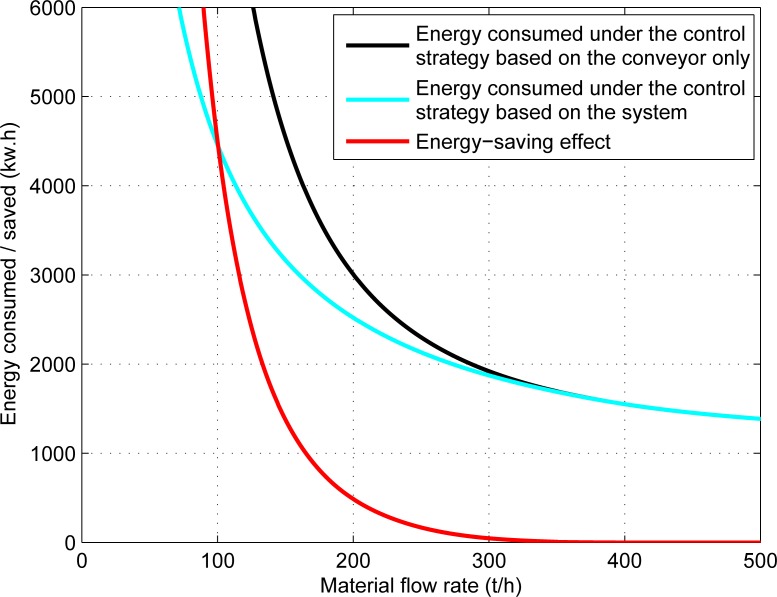
The energy consumption (energy saving) under the different control strategies.

According to Eqs (26) and (30), the belt speed of belt conveyor is directly proportional to the energy consumption of motor and frequency converter. Therefore, when 0<*M*<3.6*q*_*Gm*_*v*_*pmin*_, the energy-saving effect of this control strategy (based on the system) is significantly better than the control strategy based on the belt conveyor only. The energy-saving effect for this practical case is shown in Fig 7.

Fig 7 shows that when 0<M<386*t/h*, the energy-saving effect of this control strategy is obviously superior, and the energy-saving effect is more obvious with the decrease of material flow.

Therefore, the energy saving effect of this control strategy is better than that of running at rated speed or the control strategy based on the belt conveyor only.

### 4.3 Energy consumption of the main components of the system

**Fig 8:**
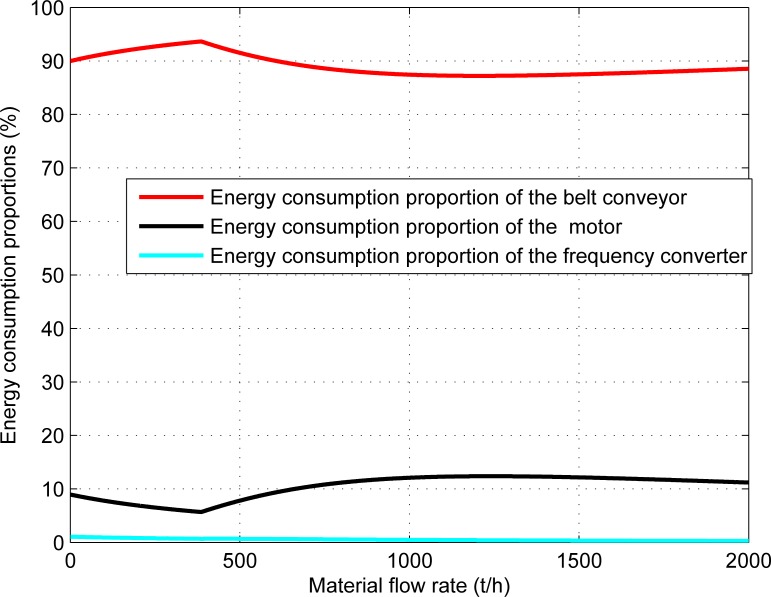
The energy consumption proportions of the main components to the total energy consumption of the system.

**Fig 9:**
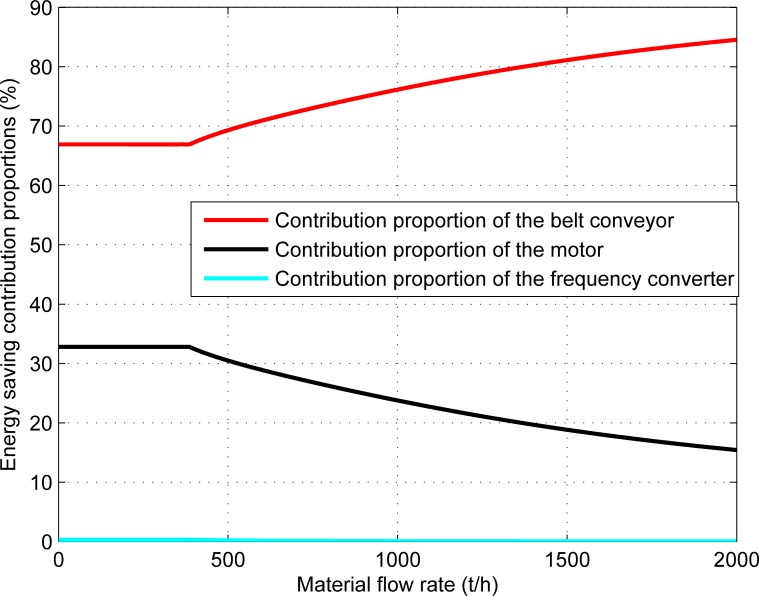
The contribution proportions of the main components to energy conservation of the system.

The energy consumption ratio of each main component of the belt conveyor system to the total energy consumption of the system and its contribution ratio to the energy-saving effect of the system under the energy-saving control strategy are shown in Figs 8 and 9 respectively.

Fig 8 shows that the energy consumption of the belt conveyor system mainly comes from the belt conveyor and motor. For a varying material flow rate, the belt conveyor accounts for about 90% of the total energy consumption of the system while the motor accounts for about 10%. The frequency converter only accounts for 0.277% to 1.071% of the total energy consumption of the system and can thus be ignored.

Fig 9 shows that the main energy-saving sources of the belt conveyor system are the belt conveyor and motor. With an increase in the material flow rate, the ratio of the contribution of the belt conveyor to system energy savings increases from 66.907% to 84.536% while the ratio of the contribution of the motor to the system energy savings decreases from 32.797% to 15.425%. The ratio of the contribution of the frequency converter to the energy savings of the system is only 0.039%–0.296% and can thus be ignored.

Therefore, the energy-saving control of the belt conveyor system should mainly focus on the belt conveyor and motor.

## Conclusion

A power consumption model of the belt conveyor system was first deduced. The model takes into account the power consumption of the motor, frequency converter, and belt conveyor and fully considers the resistance of the belt conveyor during operation. The model is therefore accurate and widely applicable. A energy-saving control strategy of the belt conveyor system based on the material flow rate was then proposed. The strategy has a good energy saving effect and effectively avoids material stockpiling. Finally, on the basis of the comprehensive analysis of the energy consumption of the main components of the belt conveyor system, the viewpoint that the energy-saving control of the belt conveyor system should mainly focus on the belt conveyor and motor was put forward.
